# Central nervous system penetration of imatinib in acute lymphoblastic leukemia: Pharmacokinetic analysis and clinical implications

**DOI:** 10.1007/s00280-026-04914-9

**Published:** 2026-06-19

**Authors:** Anna Sofie Buhl Rasmussen, Cecilie Utke Rank, Ib Jarle Christensen, Allan Weimann, Kasper Hansen, Maria Thastrup, Tianwu Yang, Trine Meldgaard Lund, Hilde Skuterud Wik, Hartmut Vogt, Ulrika Norén-Nyström, Goda Vaitkeviciene, Birgitte Klug Albertsen, Peder Skov Wehner, Bodil Als-Nielsen, Christen Lykkegaard Andersen, Kim Dalhoff, Kjeld Schmiegelow

**Affiliations:** 1 Department of Pediatrics and Adolescent Medicine, Copenhagen University Hospital, Rigshospitalet, Blegdamsvej 9, DK-2100 Copenhagen, Denmark; 2 Department of Hematology, Copenhagen University Hospital, Rigshospitalet, Copenhagen, Denmark; 3 Molecular Unit, Department of Pathology, Copenhagen University Hospital, Herlev and Gentofte Hospital, Herlev, Denmark; 4 Department of Drug Design and Pharmacology, Faculty of Health and Medical Sciences, University of Copenhagen, Copenhagen, Denmark; 5 Department of Haematology, Oslo University Hospital Rikshospitalet, Oslo, Norway; 6 Department of Biomedical and Clinical Science, Linköping University, Linköping, Sweden; 7 Crown Princess Victoria Children’s Hospital, Region Östergötland, Linköping, Sweden; 8 Department of Clinical Sciences, Pediatrics, Umeå University, Umeå, Sweden; 9 Clinic of Children’s Diseases, Institute of Clinical Medicine, Vilnius University, Vilnius, Lithuania; 10 Center for Pediatric Oncology and Hematology, Vilnius University Hospital Santaros Klinikos, Vilnius, Lithuania; 11 Department of Pediatrics and Adolescent Medicine, Aarhus University Hospital, Aarhus, Denmark; 12 Department of Clinical Medicine, Aarhus University, Aarhus, Denmark; 13 Department of Pediatric Hematology and Oncology, H. C. Andersen Children’s Hospital, Odense University Hospital, Odense, Denmark; 14 Research Unit for General Practice, Department of Public Health, University of Copenhagen, Copenhagen, Denmark; 15 Department of Clinical Pharmacology, Copenhagen University Hospital, Bispebjerg, Copenhagen, Denmark; 16 Department of Clinical Medicine, Faculty of Health and Medical Sciences, University of Copenhagen, Copenhagen, Denmark

**Keywords:** Acute lymphoblastic leukemia, Pediatric, Young adults, Imatinib, Cerebrospinal fluid, Central nervous system

## Abstract

**Purpose:**

Tyrosine kinase inhibitors (TKIs) have improved outcomes in Philadelphia chromosome-positive (Ph+) acute lymphoblastic leukemia (ALL) and are increasingly incorporated into treatment protocols of Philadelphia chromosome-like (Ph-like, ABL-class) ALL. However, central nervous system (CNS) relapse remains a significant challenge. Imatinib, a first-generation TKI, demonstrates limited CNS penetration in adults, yet data in children are sparse.

**Methods:**

This prospective, multicenter study investigated cerebrospinal fluid (CSF) and matched plasma concentrations of imatinib and its primary bioactive metabolite (N-desmethyl-imatinib) in children and young adults with Ph + or Ph-like (ABL-class) ALL. Plasma and CSF samples were analyzed with liquid chromatography tandem mass spectrometry (LC-MS/MS). Linear mixed-effects models were used to assess concentrations across compartments and over time.

**Results:**

Between January 2023 and June 2025, 32 paired plasma and CSF samples were collected from ten patients (range 1–8 samples/patient; median: 3). In total, 78% of plasma imatinib concentrations were above 1,000 ng/mL. On average, plasma imatinib concentrations were 189-fold higher than in CSF (geometric mean ratio, 95% CI: 142–249, *p* < 0.001), which was even more profound for N-desmethyl-imatinib (geometric mean ratio 273, 95% CI: 218–339, *p* < 0.001). The highest measured CSF-imatinib concentration was 63 ng/mL. During therapy, both plasma and CSF imatinib concentrations declined at an average of 0.32% per day (*p* < 0.001), resulting in stable CSF-to-plasma ratio over a median follow-up of 5.7 months (range: 0–14 months).

**Conclusion:**

The very low CSF concentrations support the rationale for alternative TKIs with improved CNS distribution in patients at risk of CNS relapse.

**Supplementary Information:**

The online version contains supplementary material available at 10.1007/s00280-026-04914-9.

## Introduction

Tyrosine kinase inhibitors (TKIs) effectively target the constitutively active ABL kinase in Philadelphia chromosome positive (Ph+) acute lymphoblastic leukemia (ALL). By blocking the ATP-binding site of BCR-ABL1, TKIs impede downstream signaling and leukemic cell proliferation [1]. The introduction of TKIs has doubled long-term survival in Ph+ ALL over the past decades [2]. Imatinib, the first-generation TKI, remains the most extensively investigated agent in this class.

A related subgroup, Philadelphia chromosome-like (Ph-like) ALL, shows a kinase-activated gene expression profile similar to Ph+ leukemia [3, 4]. With growing preclinical evidence of TKI sensitivity in Ph-like (ABL-class) disease, TKIs have now entered frontline treatment protocols for this patient population [5].

Despite therapeutic advances, central nervous system (CNS) relapse remains a significant clinical challenge in ALL [6]. The incidence of CNS involvement at diagnosis by cytospin preparations is 5–15% in both pediatric and adult ALL, with isolated CNS relapse after achieving remission occurring in approximately 5% of cases [6, 7].

The CNS presents unique challenges to pharmacological therapy due to the blood-brain barrier, which features specialized tight junctions that restrict passive diffusion of drugs from the systemic circulation into the cerebrospinal fluid (CSF), as well as active efflux transporters that may limit drug accumulation [7, 8]. Consequently, routine assessment of CNS involvement and prophylactic intrathecal chemotherapy accompanying CNS-penetrating high-dose chemotherapy remain essential components of standard treatment for ALL patients at risk for or already diagnosed with CNS leukemia [6, 7].

Despite being lipophilic, although far less than several other TKIs, imatinib demonstrates limited CNS penetration. Case reports in adult patients with Ph+ ALL or chronic myeloid leukemia (CML) in lymphoid blast crisis have documented varying CSF imatinib concentrations that are consistently lower than plasma levels, with CSF concentrations ranging from 38-fold to 175-fold below plasma concentrations [9–11]. Several mechanisms may contribute to this markedly limited CNS penetration. Preclinical models have demonstrated that P-glycoprotein-mediated efflux at the blood-brain barrier plays a central role in restricting imatinib transport into the CNS [12]. Additionally, imatinib is highly protein-bound in plasma (approximately 95%), while CSF contains substantially lower protein levels [8, 13]. Although the unbound fraction of imatinib (~ 5%) would be expected to passively diffuse into the CSF, the very low CSF concentrations observed suggest that active efflux via P-glycoprotein in brain endothelial cells markedly limits CNS exposure [8]. Consequently, the concentrations of imatinib achieved in the CSF appear to be well below those associated with effective BCR-ABL1 inhibition [10, 14, 15]. N-desmethyl-imatinib, the primary metabolite of imatinib, also demonstrates tyrosine kinase inhibitory activity (approximately 20% of the parent compound in steady-state), yet its CNS penetration has not been comprehensively characterized [16]. Importantly, data on CSF imatinib and metabolite concentrations in children are sparse. While some protocols are considering alternative TKIs such as dasatinib, which have demonstrated improved blood-brain barrier penetration [17, 18], comprehensive data on imatinib’s CNS distribution in childhood ALL is lacking.

This study aimed to investigate the relationship between CSF and plasma levels of imatinib and its active metabolite in children and young adults with Ph + or Ph-like ALL.

## Materials and methods

### Patients

This prospective, observational study included patients with Ph+ or Ph-like (ABL-class) ALL who received imatinib as part of their treatment protocol. Patients were enrolled from October 2021 and onwards, and plasma and CSF samples were collected consecutively between January 2023 and June 2025 from departments of pediatric oncology and departments of hematology in Denmark, Sweden, Norway, and Lithuania.

Patients with Ph-like (ABL-class) ALL were enrolled in the international, multicenter ALLTogether1 trial including children and young adults (aged 0–45 years) (www.clinicaltrials.gov: NTC04307576). This trial included a non-randomized intervention arm testing the addition of imatinib to standard chemotherapy for Ph-like (ABL-class) ALL. Ph+ ALL patients were excluded from the ALLTogether1 trial but could still be treated off-protocol and participate in non-randomized sub-studies. A pharmacokinetic sub-study of ALLTogether1 was conducted with separate informed consent to collect samples from patients receiving imatinib. This sub-study also included flagging of suspected non-adherence to oral imatinib therapy when plasma imatinib concentrations were below the limit of detection.

Patients with Ph+ ALL were treated according to the EsPhALL2017/COGAALL1631 protocol, a randomized, international phase III trial testing imatinib in combination with different chemotherapy backbones for patients aged 0–20 years (www.clinicaltrials.gov: NCT03007147). Sample collection from these patients was conducted under a separate Danish pharmacokinetic observational study, for which informed consent was obtained independently of the EsPhALL2017 trial.

Both protocols followed the same imatinib dosing guidelines for children (340 mg/m^2^/day with a maximum of 800 mg/day). In ALLTogether1, adults received a standard dose of 600 mg/day. Imatinib was introduced on Day 15 of induction (< 25 years of age) and Day 30 (≥ 25 years of age) and body surface area (BSA) should be calculated before each therapy phase. The EsPhaLL2017 protocol had no special dose for adults. In this protocol, imatinib was introduced at the time of study entry and dose should be recalculated at least every 12 weeks. For both protocols, imatinib dose modifications were only allowed due to severe toxicities.

Inclusion criteria for this study required collection of paired plasma and CSF samples within a two-hour interval with documented information about specific time for sampling and oral imatinib intake (dose prior to sampling). Samples collected before January 2023 were excluded due to insufficient documentation of sampling or medication times.

Plasma and CSF samples were collected prospectively from all enrolled patients at already scheduled time points for sampling according to protocol guidelines. CSF samples (1–3 mL) were obtained at lumbar puncture or intrathecal therapy infusion. Blood samples consisted of 3 mL EDTA-anticoagulated blood (minimum 1 mL for smaller children).

### Ethical approvals

This pharmacokinetic study was approved by the Scientific Ethics Committee, Capital Region of Denmark (H-22057870) and conducted in accordance with the Declaration of Helsinki (2024). Written informed consent was obtained from all patients or legal guardians prior to sample collection. The ALLTogether1 trial (EudraCT 2018-001795-38) and EsPhALL2017/COGAALL1631 (EudraCT 2017-000705-20) were approved by respective national ethics committees in participating countries.

### Sample collection and storage

Samples were sent from clinical sites to the Pediatric Oncology Research Laboratory (Bonkolab), Rigshospitalet, Copenhagen. Plasma samples were centrifuged 3,220 × g for 10 min at room temperature upon receipt. CSF samples were mixed upon receipt to ensure homogeneity. Both plasma and CSF samples were aliquoted into Micronic tubes and stored at -80 °C until analysis.

Each sample was accompanied by a requisition form from the clinical site containing information on the time of blood and CSF sample collection, time of oral imatinib intake, imatinib dose, and patient weight and height.

### Imatinib and metabolite detection in plasma

#### Sample preparation

Frozen plasma samples were thawed at room temperature and vortexed prior to analysis. To a 100 µL sample/standard/quality control was added 40 µL of internal standard solution and the mixture was vortexed. Subsequently, 1 mL of ethyl acetate was added followed by vortex mixing. The samples were then centrifuged at 13,500 × g for 5 min. The supernatant (250 µL) was transferred to Micronic tubes and evaporated to dryness. The residue was reconstituted in 500 µL of 15% acetonitrile containing 0.1% formic acid and vortexed.

#### Preparation of standards

Matrix-matched calibration standards were prepared by spiking known amounts of imatinib and N-desmethyl-imatinib into blank human plasma. The standards were extracted following the same procedure as the plasma samples. The calibration curve for imatinib was prepared over the range 94–9,636 ng/mL, and the validated lower limit of quantification (LLOQ) was established at 94 ng/mL based on the lowest calibration standard analyzed in duplicate on two datasets. For N-desmethyl-imatinib, calibration standards were prepared over the range 50–5,000 ng/mL. The validated LLOQ was established at 50 ng/mL based on the lowest calibration standard analyzed in duplicate.

The limit of detection (LOD) was determined based on signal-to-noise criteria. For imatinib, the lowest QC sample (74 ng/mL) showed a signal-to-noise ratio greater than 3 and was therefore considered above the detection limit. For N-desmethyl-imatinib, the lowest injected standard likewise showed a signal-to-noise ratio exceeding 3. These values are consistent with previously reported method validation data.

#### LC-MS/MS analysis

Analysis was performed using a Sciex Qtrap 6500+ mass spectrometer coupled to a Sciex ExionLC UHPLC system (Sciex, Framingham, MA, USA). Chromatographic separation was achieved on a Waters HSS T3 column (50 × 2.1 mm, 1.8 μm) (Waters, Milford, MA, USA) maintained at 40 °C.

Eluent A consisted of 50 mM ammonium formate (pH 3.2) and Eluent B was acetonitrile. The gradient program started at 4.5% B, held until 0.15 min, then increased to 31.5% B at 0.16 min, followed by a linear increase to 40.5% B at 0.8 min, and further to 85.5% B at 2.7 min, where it was held until 3.9 min. B was then decreased to 4.5% at 4.02 min and held until 5.0 min. The flow rate was 400 µL/min throughout.

Ions were generated by electrospray ionization in positive ion mode and data were recorded in multiple reaction monitoring mode. The injection volume was 0.2 µL for each plasma sample and standard. Between injections of each sample/standard, 10 µL of 2-propanol was injected to reduce carryover.

### Imatinib and metabolite detection in CSF

#### Sample preparation

Frozen CSF samples were thawed at room temperature prior to analysis. To 100 µL of CSF was added 10 µL of internal standard solution. The mixture was vortexed and left for 15 min to equilibrate before protein precipitation with 500 µL of acetonitrile. The samples were then centrifuged at 3,220 × g for 15 min.

#### Preparation of standards

Matrix-matched calibration standards were prepared by spiking 10 µL of solution containing known amounts of imatinib and N-desmethyl-imatinib into 90 µL of blank CSF. Protein precipitation and addition of internal standard solution were performed following the same procedure as the CSF samples. Imatinib was calibrated in the range of 1–127 ng/mL and N-desmethyl-imatinib in the range of 0.16–20 ng/mL. Based on the calibration data, the LLOQ was established at 1 ng/mL for imatinib and 0.16 ng/mL for N-desmethyl-imatinib, corresponding to the lowest calibration standards analyzed in duplicate across two datasets. The LOD was determined using a signal-to-noise criterion. For imatinib, the lowest calibration standard (1 ng/mL) produced a signal-to-noise ratio greater than 3. For N-desmethyl-imatinib, the lowest injected standard (0.16 ng/mL) likewise exceeded a signal-to-noise ratio of 3. These observations are consistent with previously established method validation data.

#### LC-MS/MS analysis

The LC-MS/MS conditions were identical to those described for the plasma samples, except that 1 µL of supernatant from each sample and standard was injected.

#### Quality control

All plasma and CSF samples were measured in duplicate, and the mean values were used in the analyses. Because CSF sample volumes were limited, it was not feasible to prepare a full set of CSF quality-control samples for constructing a control chart. However, analytical performance for CSF samples was assessed based on replicate measurements and calibration data within the concentration range of the study.

The intra-assay variation for CSF, expressed as the coefficient of variation (CV%) between duplicate injections, ranged from 0% to 7%, which is well below commonly accepted bioanalytical criteria (≤ 15%) [19]. In combination with the established calibration curve and LLOQ, these results support that the method provides accurate and quantitative measurements for both imatinib and N-desmethyl-imatinib across the studied concentration range.

For plasma, routine internal quality-control procedures were applied throughout the study period, including the use of a control chart to monitor inter-assay variation. Proficiency-test samples for imatinib in serum were analyzed at regular intervals to verify external analytical performance. Proficiency-test samples were not available for imatinib in CSF, nor for N-desmethyl-imatinib in either plasma or CSF.

### Statistical analyses

Descriptive statistics are presented as medians with minimum and maximum values, and interquartile ranges (25th and 75th percentiles) for continuous variables and as counts with percentages for categorical variables. Patient age was calculated at the time of study initiation. BSA calculation was done by Mosteller’s square root method [20].

CNS penetration was defined as the CSF-to-plasma concentration ratio at steady-state, estimated as the geometric mean ratio by back-transforming the sample type coefficient from the log-scale model. To assess imatinib and N-desmethyl-imatinib concentrations across sample types (plasma and CSF) and over time, linear mixed-effects models were employed. Concentration values were log-transformed to achieve normality and stabilize variance. Samples were collected during treatment at varying times post-dose. While this approach does not allow full pharmacokinetic characterization, it is appropriate for the study objective of comparing compartmental concentration ratios and assessing their stability over the treatment course. These models incorporated a random intercept term for each patient to account for within-subject variability. Fixed effects included sample type, time from first sample, time since last oral imatinib intake, and BSA-adjusted dose. Model validation was done to investigate the normality of residuals and random effects.

The relationship between imatinib and metabolite concentrations in plasma and CSF was assessed using linear regression. Results are presented as regression coefficients with corresponding 95% confidence interval (CI) and the adjusted R^2^ values.

Two-sided p-values < 0.05 was considered statistically significant. All statistical analyses were performed using R version 4.4.2 (R Core Team, 2024) in RStudio (Posit team, 2025). Linear mixed-effects models were fitted using lme4 [21] and lmerTest [22] packages.

## Results

Ten patients with ALL were included in this study (Table 1). The cohort consisted of seven males (70%) and three females (30%) with median age at diagnosis of 10 years (range: 2–22 years). The cohort was evenly distributed between Ph-like (ABL-class) ALL (*n* = 5) and Ph+ ALL (*n* = 5). Median time from diagnosis to first sample collection was 9.9 months (range: 2.2–21.3 months) and from imatinib therapy initiation to first sample was 9 months (range: 0.5–24.1 months). One patient underwent hematopoietic stem cell transplantation during the sampling period but resumed imatinib treatment according to protocol. No patients had CNS involvement at the time of diagnosis.

**Table 1:** Baseline demographic data

Characteristics	*N* = 10
Age at diagnose (years), median (min, Q1, Q3, max)	10 (2, 5.8,10.8, 22)
Time from diagnose to first sample (months), median (min, Q1, Q3, max)	9.9 (2.2, 5.3, 15.1, 21.3)
BSA at first sample (m^2^), median (min, Q1, Q3, max)	1.1 (0.6, 0.8, 1.5, 2.1)
Sex, *N*(%)	
Male	7 (70)
Subtype ALL, *N*(%)	
Ph-like	5 (50)
Ph+	5 (50)
Imatinib dose at first sample (mg), median (min, Q1, Q3, max)	275 (194, 200, 400, 600)
Plasma imatinib concentration (ng/mL), median (min, Q1, Q3, max) (*N* = 32)	1,594 (219, 1,089, 2,634, 6,253)
Plasma metabolite concentration (ng/mL), median (min, Q1, Q3, max) (*N* = 23)	484 (118, 364, 596, 1,278)
CSF imatinib concentration (ng/mL), median (min, Q1, Q3, max) (*N* = 32)	8.7 (0.5, 5.7, 14.6, 63.4)
CSF metabolite concentration (ng/mL), median (min, Q1, Q3, max) (*N* = 32)	1.9 (0.5, 1.1, 2.8, 12.1)

ALL: Acute lymphoblastic leukemia, BSA: Body surface area, CSF: cerebrospinal fluid, Ph+: Philadelphia chromosome positive, Ph-like: Philadelphia chromosome-like, Q1: 25th percentile, Q3: 75th percentile

A total of 32 paired plasma and CSF samples were collected for analysis (range: 1–8 samples per patient). Imatinib concentrations were measured in all samples, while N-desmethyl-imatinib (metabolite) concentrations were available for 23 plasma samples and all 32 CSF samples due to initial technical limitations with the plasma metabolite assay. One CSF-imatinib sample were below LLOQ.

The median imatinib dose was 292 mg/m^2^/day (range: 150–358 mg/m^2^/day) but varied for the individual patient during the study (Online Resource 1). Substantial variability was observed in the time from oral imatinib intake to sample collection with a median of 16 h (range: 2.8–24.5 h). Nearly half (48.4%) of all samples were collected 15–20 h after medication intake (Online Resource 2). CSF imatinib concentrations in relation to time from last oral dose are shown in Online Resource 3, demonstrating considerable variability.

### Imatinib concentrations

**Fig. 1:**
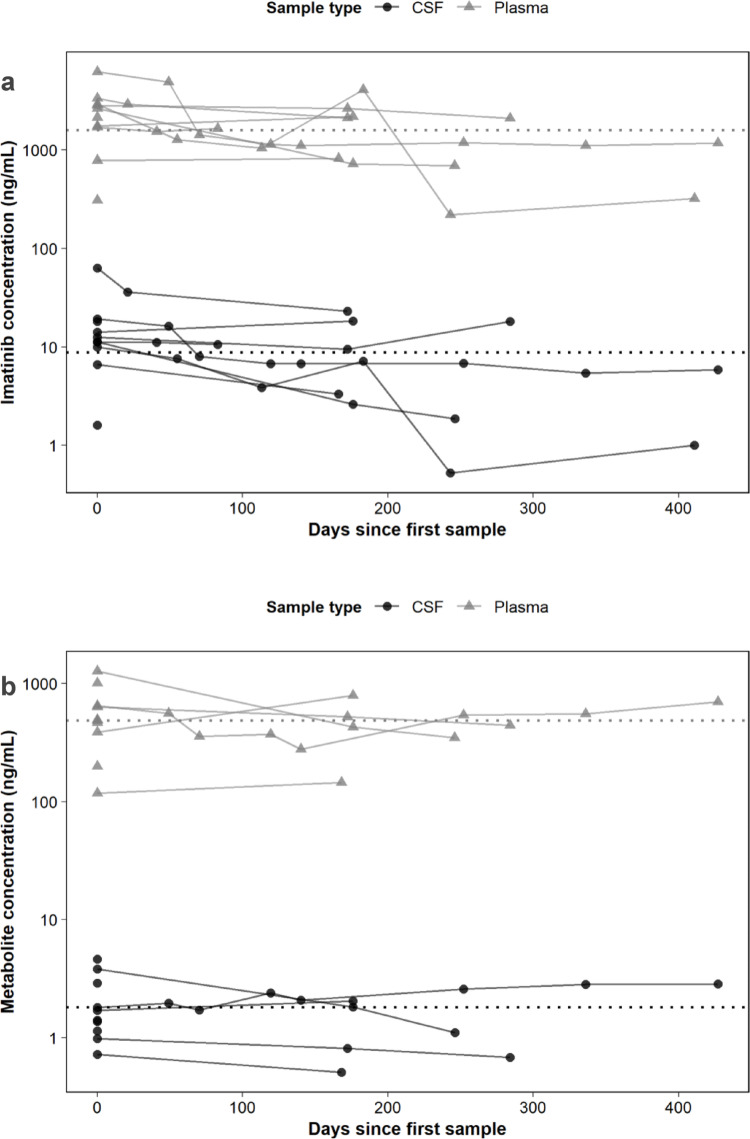
Longitudinal imatinib and metabolite concentrations by compartment. Individual patient trajectories showing (**a**) imatinib and (**b**) N-desmethyl-imatinib concentrations in plasma and cerebrospinal fluid (CSF) plotted against days since first sample collection. Each line connects sequential samples from the same patient. Plasma samples are indicated by triangular markers, and CSF samples by circular markers. Dashed horizontal lines indicate median concentrations. Concentrations are displayed on a logarithmic scale

**Fig. 2:**
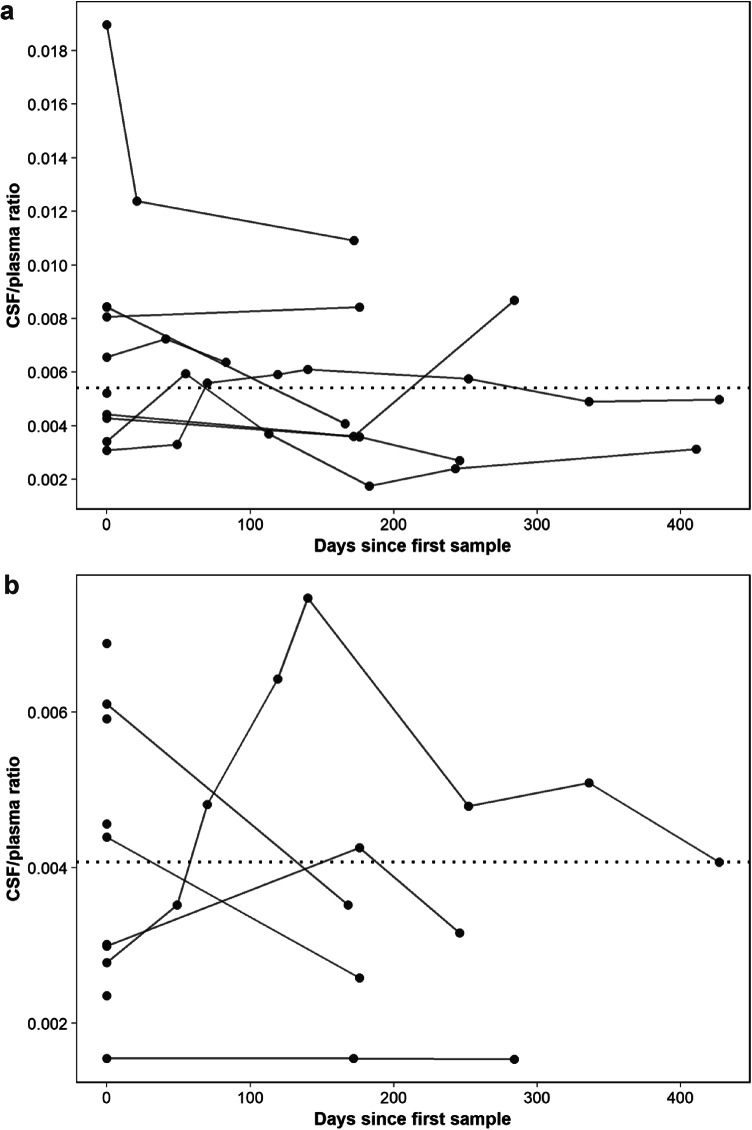
Imatinib and metabolite CSF/plasma ratios over time. Individual patient trajectories for (**a**) imatinib and (**b**) N-desmethyl-imatinib CSF-to-plasma concentration ratios plotted against days since first sample collection. Each line connects sequential samples from the same patient. Points represent individual observations. Dashed horizontal lines indicate median ratios

Median imatinib plasma concentration was 1,594 ng/mL (Table 1), with a central 75% range of 720–2,964 ng/mL. Overall, 25 of 32 plasma samples (78%) were above 1,000 ng/mL. Assessing linear mixed-effects models, plasma imatinib concentrations were substantially higher than CSF concentrations (mean 189-fold difference, 95% CI: 142–249, *p* < 0.001). Both compartments demonstrated a significant decline over the treatment course (0.32% per day, 95% CI: 0.19–0.44% per day, *p* < 0.001), corresponding to approximately a 27% reduction over 100 days. The primary model demonstrated excellent fit, with fixed effects explaining 90.2% of variance (marginal R²) and the full model including patient-level random effects explaining 95.9% of variance (conditional R²). Imatinib concentrations in plasma and CSF over time are visualized in Fig. 1a, while imatinib CSF/plasma ratios are displayed in Fig. 2a. The distribution of imatinib CSF/plasma ratios across all samples are presented in Online Resource 4.

A supplementary model including an interaction term between sample type and time from first sample revealed no significant difference in the rate of decline between plasma and CSF (*p* = 0.31), indicating that the CSF-plasma concentration ratio did not change throughout follow-up, consistent with a stable equilibrium between compartments.

### Metabolite concentrations

Plasma metabolite concentrations were even more elevated compared to CSF than the parent compound (mean 273-fold difference, 95% CI: 218–339, *p* < 0.001). Unlike imatinib, metabolite concentrations showed no significant decline over time (0.01% per day, 95% CI: -0.12%–0.09% per day, *p* = 0.91), suggesting stable metabolite levels throughout the study period. A supplementary model including an interaction term between sample type and time from first sample found no significant difference between plasma and CSF metabolites over time (*p* = 0.78). Temporal imatinib metabolite concentrations in plasma and CSF are presented in Fig. 1b, and ratio between metabolite in CSF to plasma are displayed in Fig. 2b. The distribution of metabolite CSF/plasma ratios across all samples are presented in Online Resource 4.

### Sensitivity analysis

To assess the robustness of findings and potential influence of later time points, sensitivity analyses were conducted restricting datasets to observations within the first 300 days of treatment. For imatinib, this included 58 of 64 observations. Results remained consistent with the primary analysis. For metabolites, the sensitivity analysis included 42 of 46 observations. The dataset showed a non-significant trend toward decline (0.12% per day, 95% CI: -0.01%–0.26% per day, *p* = 0.10), which remained consistent with the primary analysis showing stable metabolite levels. These findings confirm that results were not substantially influenced by the inclusion of longer follow-up data for either compound.

A sensitivity analysis excluding the one CSF-imatinib sample below the LLOQ yielded consistent results (data not shown).

### Correlation between imatinib and metabolite concentrations

Linear regression analyses revealed significant positive associations between compartments and between parent drug and metabolite (Online Resource 5). Plasma imatinib concentrations strongly predicted CSF imatinib concentrations (adjusted R^2^ = 0.74, *p* < 0.001), while the association was moderate for the metabolite (adjusted R^2^ = 0.40, *p* < 0.001). Within compartments, imatinib and metabolite concentration were moderately associated in plasma (adjusted R^2^ = 0.59, *p* < 0.001) and weakly correlated in CSF (adjusted R^2^ = 0.22, *p* = 0.004).

## Discussion

This is the first prospective multicenter study reporting imatinib and N-desmethyl-imatinib concentrations of CSF in children and young adults with ALL. We found limited CNS penetration of both imatinib and its metabolite, with CSF concentrations substantially lower than plasma concentrations throughout the study period. The CSF-to-plasma concentration ratios remained stable over time and did not vary significantly with time since oral dosing.

Our findings exceed previous case reports in adult patients with CML in lymphoid crisis or Ph+ ALL, which demonstrated CSF-to-plasma ratios ranging from 38-fold to 175-fold lower in CSF (based on daily imatinib 200–600 mg) [9–11, 23]. We observed a ratio of plasma concentrations approximately 189-fold higher than CSF concentrations. Only one previous study reported metabolite values and found a similar ratio to the parent drug [11]. Notably, our longitudinal data suggested divergent temporal patterns between imatinib and its metabolite. While imatinib concentrations declined significantly over time in both compartments, metabolite concentrations remained stable. However, metabolite data were not available for all plasma samples, limiting our ability to draw robust conclusions. The divergence could suggest time-dependent changes in drug metabolism, potentially through CYP3A4 enzyme induction leading to enhanced conversion of parent drug to metabolite, though further investigation with complete metabolite data would be needed to confirm this hypothesis.

The poor CNS penetration observed in this study raises concerns about the adequacy of imatinib in the prevention of CNS relapse in childhood ALL. Several studies have reported CNS relapses in patients achieving complete hematologic remission [23, 24], including children with CML in lymphoid blast crisis [25]. While our study was not designed to evaluate clinical outcomes, the consistently low CSF concentrations suggest limited CNS exposure. For context, 78% of the plasma samples in the study exceeded 1,000 ng/mL, a threshold associated with higher molecular response rates in adult CML, although not validated in ALL or in children. However, the target imatinib concentration required for therapeutic efficacy in the CNS remains unknown [14, 15]. All CSF concentrations in our study (median 8.7 ng/mL; maximum 63.4 ng/mL) were below 125 ng/mL, i.e., well below concentrations reported to inhibit BCR-ABL1 activity in vitro [15, 26]. Although the clinical relevance of these thresholds for CNS disease remains uncertain, this finding supports the notion of limited pharmacologically active exposure in the CNS.

Strategies to increase CSF imatinib concentrations are challenged given the possible dose-limiting toxicities (e.g., myelosuppression) during dose escalation and the local irritant properties of imatinib when considering intrathecal administration [15]. These findings support the rationale for newer treatment protocols incorporating dasatinib, which demonstrates superior CNS penetration compared to imatinib. While the use of dasatinib in T-ALL appears promising, further pediatric data are needed [27].

We observed substantial variability in imatinib dosing with a median dose of 292 mg/m^2^/day, which was lower than the protocol guidelines. Furthermore, we noted a trend toward declining imatinib concentrations in both plasma and CSF compartments. This decline could reflect multiple factors, including suboptimal medication adherence, which is not uncommon in this patient population, or upregulation of CYP3A4 enzymatic pathways resulting in an increased metabolic turnover. Additionally, as suggested by our dose trajectories, failure to appropriately increase doses as children grow may have contributed to declining concentrations. Both included treatment protocols allow dose modifications due to severe toxicities; however, our study lacked detailed toxicity data to determine whether dose reductions were clinically justified.

The high variability in both plasma and CSF imatinib concentrations observed in this study likely reflects multiple factors including inter-individual pharmacokinetic differences, suboptimal adherence, or concomitant medications affecting imatinib metabolism. Neither BSA-normalized dose nor time since last dose were significantly associated with imatinib concentration in our mixed-effects model. However, these findings may primarily reflect that patient-level random effects absorbed most between-patient variation in typical dosing and sampling patterns, while within-patient variation in these factors was limited—most patients maintained relatively stable doses and samples were obtained at consistent post-dose intervals across visits. Therefore, these findings do not imply that dose or sampling time are unimportant for imatinib concentration monitoring. Our pragmatic sampling approach during scheduled clinical visits, as opposed to a systematically designed study to capture peak or trough concentrations, limits our ability to fully characterize the impact of sampling time on measured drug levels.

This study has several limitations. The small sample size of ten patients, though reflective of the rarity of pediatric Ph+ and Ph-like ALL, limits generalizability. We lacked information on concomitant medications, particularly CYP3A4 inducers or inhibitors, as well as renal/hepatic function status, which could significantly affect imatinib concentrations. Additionally, we did not have detailed toxicity data to determine whether observed dose reductions were clinically appropriate. Finally, the study was not designed to evaluate associations between CSF imatinib concentrations and clinical outcomes such as CNS relapse or survival.

## Conclusion

This multicenter study provides prospective data on imatinib CNS penetration in children and young adults with ALL. Our findings demonstrate consistently poor CSF penetration that does not improve over time, supporting the need for alternative TKIs with better CNS distribution in this population. Furthermore, our results underscore the importance of vigilant dose monitoring and adjustment in growing children receiving BSA-based dosing regimens.

## Supplementary Information

Below is the link to the electronic supplementary material.

Supplementary Material 1

## Data Availability

The data that support the findings of this study are available from the corresponding author upon reasonable request.
