# Supplementary material for: Central nervous system penetration of imatinib in acute lymphoblastic leukemia: Pharmacokinetic analysis and clinical implications
Source: Cancer Chemother Pharmacol. 2026 Jun 19;96(1):65. doi: 10.1007/s00280-026-04914-9 (PMC13279477; doi:10.1007/s00280-026-04914-9)
Supplement: Supplementary file 1 — Supplementary Material 1 [file 280_2026_4914_MOESM1_ESM.docx]

**Online Resource**


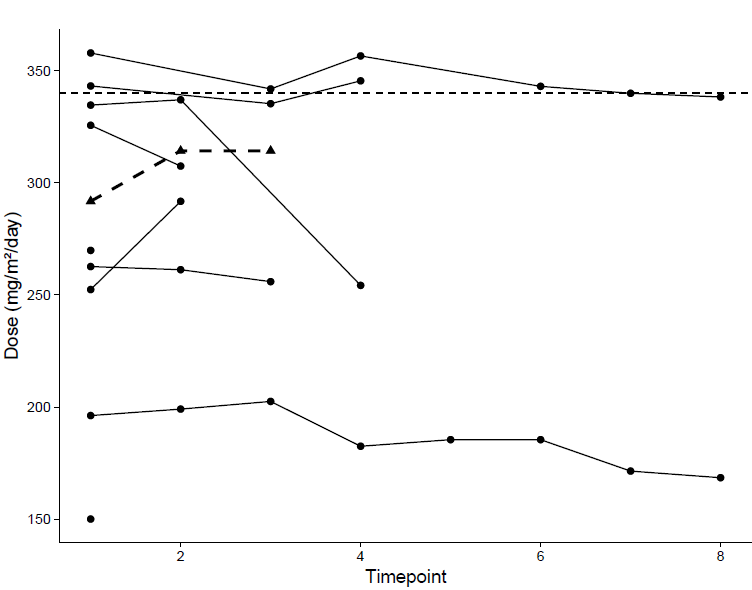


**Fig. 1 Imatinib dosing over time.** Imatinib dose trajectories for individual patients over sequential sampling timepoints. Each timepoint represent the nth sample collected from each patient (timepoint 1 = first sample, timepoint 2 = second sample, etc.), regardless of the actual time elapsed between samples. Time intervals between consecutive timepoints vary both within and between patients. The single adult patient (aged 22 years), who received a standard fixed dose of 600 mg daily, is indicated by a dashed line with triangular markers. The dashed horizontal line indicates the protocol guideline of 340 mg/m^2^/day for pediatric patients. Only 28.1% of samples were within ±10 mg/m^2^ of the guideline dose


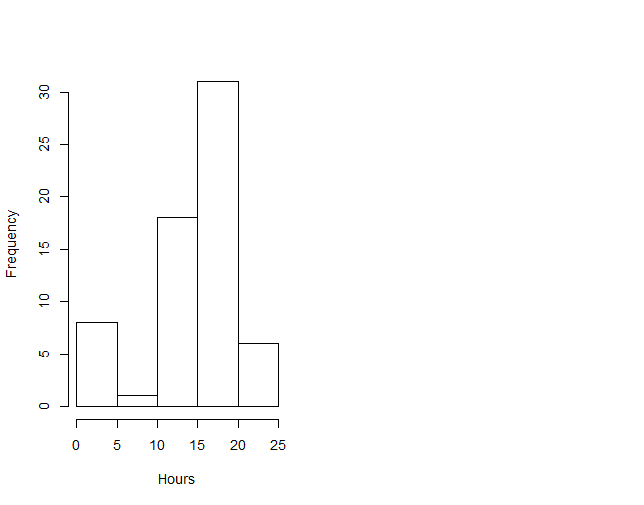


**Fig. 2 Time from medication intake to sample collection.** Frequency distribution of hours between oral imatinib intake and sample collection. Each bar represents the number of samples collected within that time window across all 64 samples


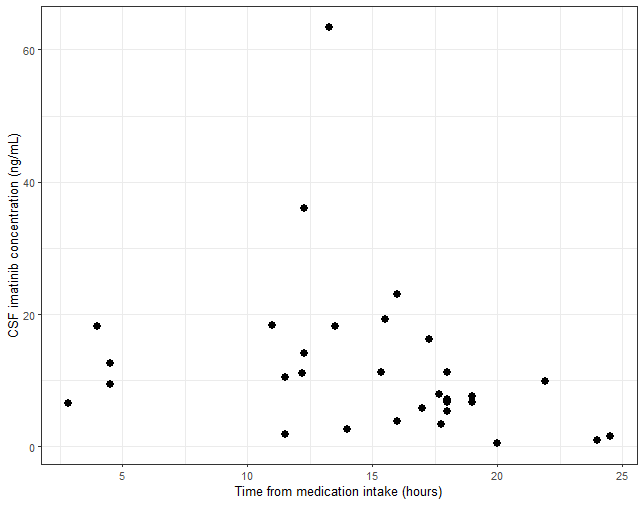


Fig. 3 CSF imatinib concentration in relation to time from last oral dose. Scatter plot showing cerebrospinal fluid (CSF) imatinib concentrations (ng/mL) versus time from last oral dose (hours) for all samples. Each point represents an individual sample


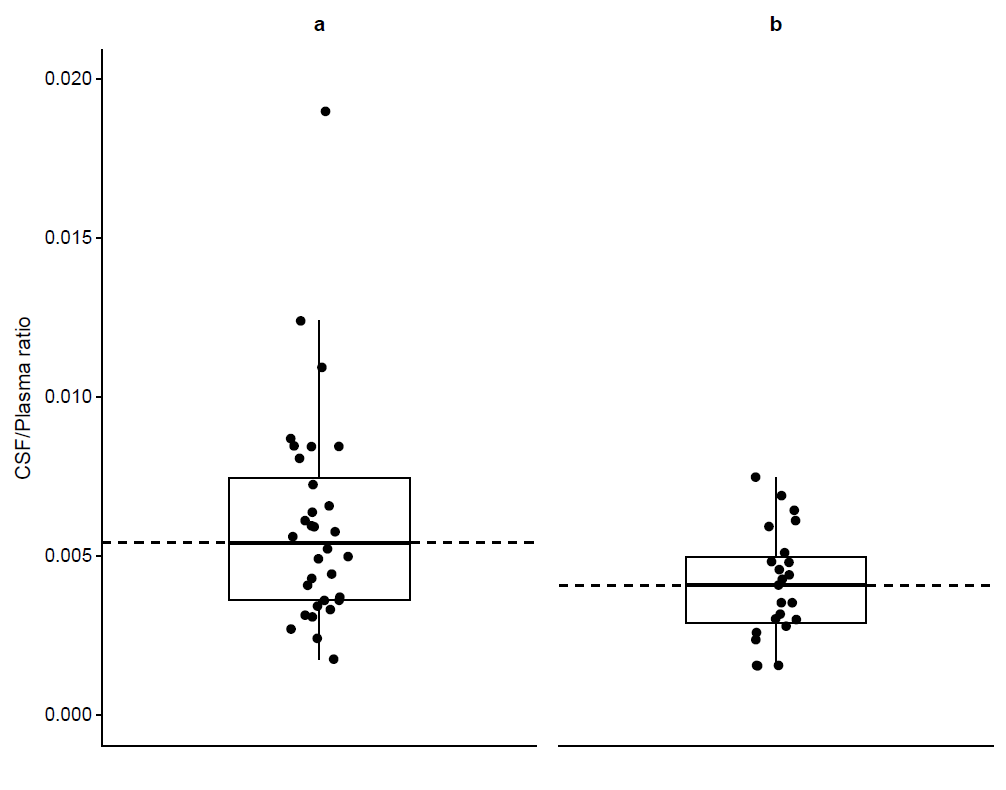


**Fig. 4** **CSF/plasma concentration ratios for imatinib and metabolite.** Box plots display the distribution of CSF-to-plasma ratios for imatinib (a) and N-desmethyl-imatinib (b). Boxes represent the interquartile range, with the median indicated by a solid line within each box; whiskers extend to the most extreme data points within 1.5× the interquartile range. Individual data points are shown as solid black markers. Dashed horizontal lines indicate median values.


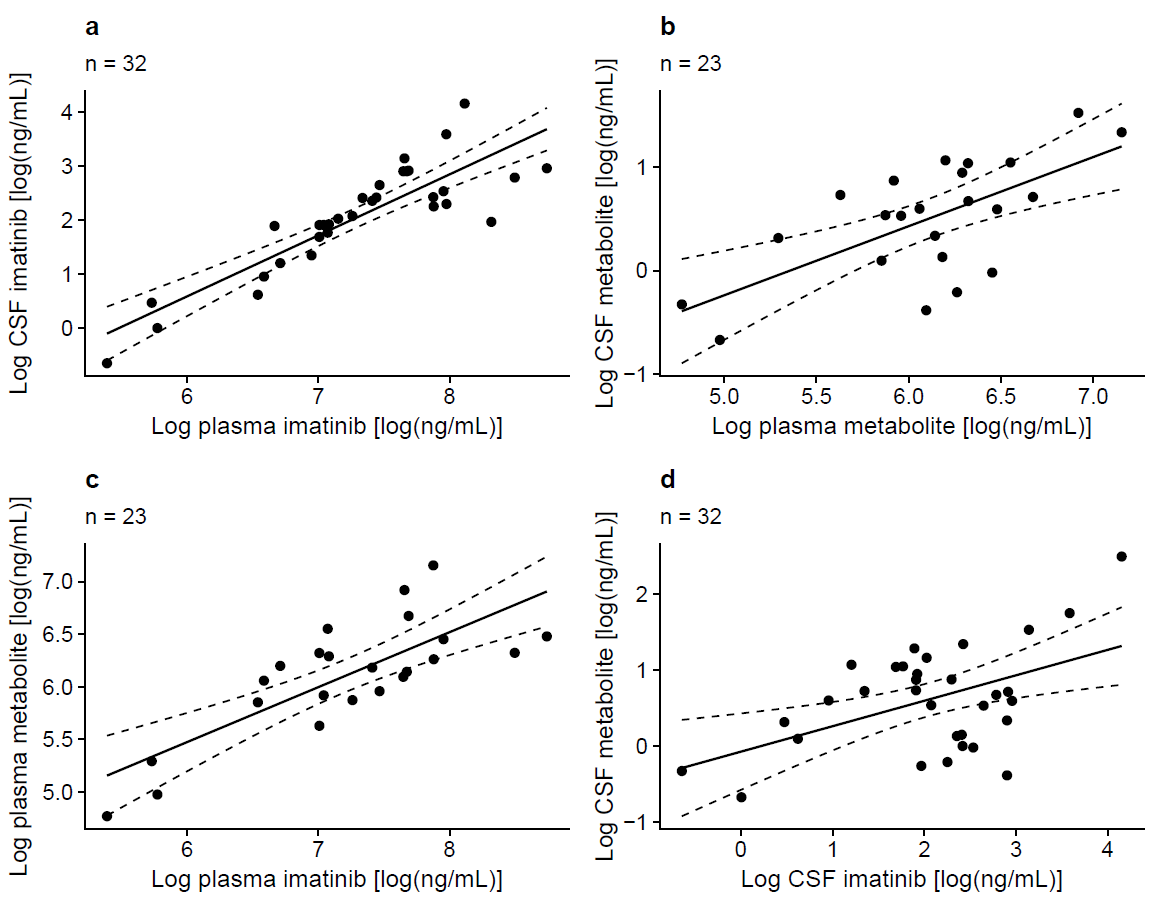


**Fig. 5 Relationship between log-transformed imatinib and metabolite concentrations in paired plasma and cerebrospinal fluid (CSF) samples.** Solid lines represent regression fits with 95% confidence intervals shown as dashed lines. Data points are displayed as solid black circles. Panel a: Plasma vs CSF imatinib (n=32). Panel b: Plasma vs CSF metabolite (n=23). Panel c: Plasma imatinib vs plasma metabolite (n=23). Panel d: CSF imatinib vs CSF metabolite (n=32)
